# [3,5-Bis(benz­yloxy)phen­yl]methanol

**DOI:** 10.1107/S1600536809010757

**Published:** 2009-04-08

**Authors:** Pei-Hua Zhu, Zi-Zhen Ni, Chun-Hui Dong, Yan-Fang Zhao, Qin Wei

**Affiliations:** a School of Chemistry and Chemical Engineering, University of Jinan, Jinan 250022, People’s Republic of China

## Abstract

In the title compound, C_21_H_20_O_3_, the two terminal phenyl rings are each approximately perpendicular to the central benzene ring, making dihedral angles of 84.40 (16) and 75.12 (15)°. The H atom of the hydr­oxy group is disordered over two positions with equal occupancies. The mol­ecules are linked by O—H⋯O hydrogen bonds, forming a chain along the *a* axis.

## Related literature

For related compounds, see: Rheiner & Seebach (1999 ▶); Pan *et al.* (2005 ▶); Xiao *et al.* (2007 ▶). For the synthesis, see: Hawker & Fréchet (1990 ▶).
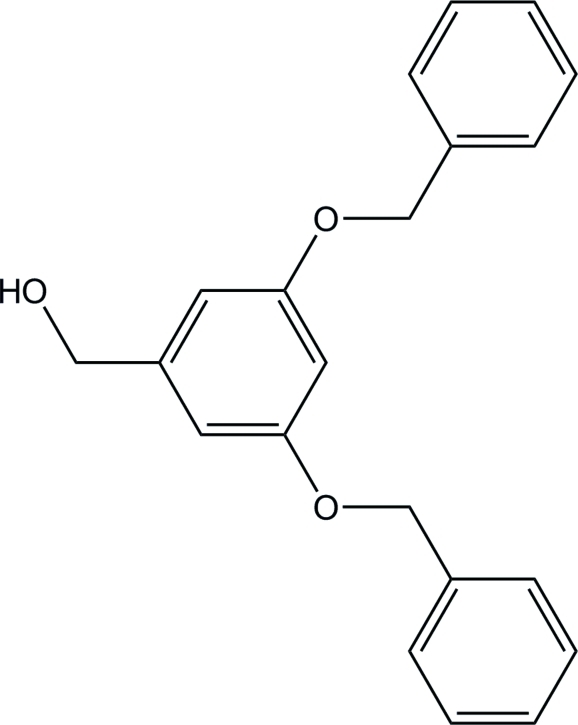


## Experimental

### 

#### Crystal data

C_21_H_20_O_3_
*M* *_r_* = 320.37
Triclinic, 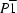

*a* = 4.8555 (6) Å
*b* = 12.2442 (18) Å
*c* = 15.017 (2) Å
α = 74.049 (1)°
β = 83.293 (1)°
γ = 89.733 (2)°
*V* = 852.2 (2) Å^3^
*Z* = 2
Mo *K*α radiation
μ = 0.08 mm^−1^
*T* = 298 K
0.46 × 0.16 × 0.15 mm

#### Data collection

Bruker APEXII CCD area-detector diffractometer
Absorption correction: multi-scan (**SADABS**; Bruker, 2001 ▶) *T*_min_ = 0.963, *T*_max_ = 0.988
4355 measured reflections
2909 independent reflections
1563 reflections with *I* > 2σ(*I*)
*R*_int_ = 0.035

#### Refinement

*R*[*F* ^2^ > 2σ(*F* ^2^)] = 0.057
*wR*(*F* ^2^) = 0.168
*S* = 0.99
2909 reflections
217 parameters
H-atom parameters constrained
Δρ_max_ = 0.37 e Å^−3^
Δρ_min_ = −0.21 e Å^−3^

### 

Data collection: *APEX2* (Bruker, 2004 ▶); cell refinement: *SAINT-Plus* (Bruker, 2004 ▶); data reduction: *SAINT-Plus*; program(s) used to solve structure: *SHELXS97* (Sheldrick, 2008 ▶); program(s) used to refine structure: *SHELXL97* (Sheldrick, 2008 ▶); molecular graphics: *SHELXTL* (Sheldrick, 2008 ▶); software used to prepare material for publication: *SHELXTL*.

## Supplementary Material

Crystal structure: contains datablocks global, I. DOI: 10.1107/S1600536809010757/is2400sup1.cif

Structure factors: contains datablocks I. DOI: 10.1107/S1600536809010757/is2400Isup2.hkl

Additional supplementary materials:  crystallographic information; 3D view; checkCIF report

## Figures and Tables

**Table 1:** Hydrogen-bond geometry (Å, °)

*D*—H⋯*A*	*D*—H	H⋯*A*	*D*⋯*A*	*D*—H⋯*A*
O3—H3⋯O3^i^	0.87	1.98	2.791 (4)	155
O3—H3′⋯O3^ii^	0.86	1.95	2.805 (5)	179

Symmetry codes: (i) 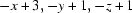; (ii) 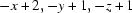.

**Fig. 1.**
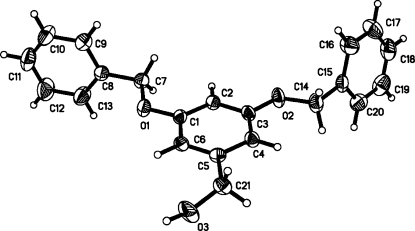
The molecular structure, with atom labels and 25% probability displacement ellipsoids for non-H atoms.

## supplementary crystallographic information

### Comment

Dendrimers are three-dimensional hyperbranched macromolecules that provide well defined nanoscopic objects at the single molecular level. Recent studies on dendritic macromolecules have extended the scope of research from synthesis to applications for catalysts, photoactive and electronic materials, medicinal and biomedical materials, and other functional materials. As a part of our structural investigations on dendritic macromolecules, the single-crystal X-ray diffraction study on the title compound was carried out.

In the title compound, the bond lengths and angles are within the normal ranges. Each unit cell of the title compound contains two molecules like other analogues reported before (Pan *et al.*, 2005; Rheiner & Seebach, 1999; Xiao *et al.*, 2007). It is worth noting that the dihedral angles between the central benzene ring and the two peripheral phenyl rings are 84.40 (16) and 75.12 (15)°. Probably because of the effects of substitution of the central benzene ring, the dihedral angels of the title compound are different from ones reported (Xiao *et al.*, 2007). The O—CH_2_ bonds lie in the plane of the central phenyl ring and adopt a *syn*, *anti* conformation (Pan *et al.*, 2005). The crystal structure is stabilized by intermolecular hydrogen bonds.

### Experimental

Benzylbromide (10.0 g, 58 mmol), 3,5-dihydroxybenzyl alcohol (4.10 g, 129 mmol), 18-crown-6-ether (1.54 g, 5.8 mmol) and potassium carbonate (16 g, 578.6 mmol) were suspended in 500 ml of 2-butanone under nitrogen atmosphere. The mixture was heated under reflux for 48 h. Upon completion of the reaction, the solvent was evaporated and the water and dichloromethanewere added to the residue. Conventional workup and purification by silica-gel column chromatography (eluent: dichloromethane) yielded 5.6 g of the title compound (60%) as a white needle (Hawker & Fréchet, 1990). Single crystals suitable for X-ray study were grown by diffusion method [dichloromethane/*n*-hexane (1:4 *v*/*v*)] at room temperature.

### Refinement

H atoms bound to carbon were refined using a riding model, with C—H = 0.93 Å and *U*_iso_(H) = 1.2*U*_eq_ (C) for aromatic atoms, and C—H = 0.97 Å and *U*_iso_(H) = 1.2*U*_eq_ (C) for methylene atoms. The H atom of the OH group was found to be disordered over two positions with approximately equal occupancies from a difference Fourier map. The positions were then constrained, with *U*_iso_(H) = 1.2*U*_eq_(O), and with equal occupancies.

### Crystal data

**Table:**

C_21_H_20_O_3_	*Z* = 2
*M**_r_* = 320.37	*F*(000) = 340
Triclinic, *P*1	*D*_x_ = 1.249 Mg m^−^^3^
Hall symbol: -P 1	Mo *K*α radiation, λ = 0.71073 Å
*a* = 4.8555 (6) Å	Cell parameters from 1252 reflections
*b* = 12.2442 (18) Å	θ = 2.5–23.9°
*c* = 15.017 (2) Å	µ = 0.08 mm^−^^1^
α = 74.049 (1)°	*T* = 298 K
β = 83.293 (1)°	Needle, colorless
γ = 89.733 (2)°	0.46 × 0.16 × 0.15 mm
*V* = 852.2 (2) Å^3^	

### Data collection

**Table:**

Bruker APEXII CCD area-detector diffractometer	2909 independent reflections
Radiation source: fine-focus sealed tube	1563 reflections with *I* > 2σ(*I*)
graphite	*R*_int_ = 0.035
φ and ω scans	θ_max_ = 25.0°, θ_min_ = 1.4°
Absorption correction: multi-scan (*SADABS*; Bruker, 2001)	*h* = −5→5
*T*_min_ = 0.963, *T*_max_ = 0.988	*k* = −14→13
4355 measured reflections	*l* = −17→17

### Refinement

**Table:**

Refinement on *F*^2^	Primary atom site location: structure-invariant direct methods
Least-squares matrix: full	Secondary atom site location: difference Fourier map
*R*[*F*^2^ > 2σ(*F*^2^)] = 0.057	Hydrogen site location: inferred from neighbouring sites
*wR*(*F*^2^) = 0.168	H-atom parameters constrained
*S* = 0.99	*w* = 1/[σ^2^(*F*_o_^2^) + (0.0808*P*)^2^] where *P* = (*F*_o_^2^ + 2*F*_c_^2^)/3
2909 reflections	(Δ/σ)_max_ = 0.001
217 parameters	Δρ_max_ = 0.37 e Å^−^^3^
0 restraints	Δρ_min_ = −0.21 e Å^−^^3^

### Special details

**Table:**

Geometry. All e.s.d.'s (except the e.s.d. in the dihedral angle between two l.s. planes) are estimated using the full covariance matrix. The cell e.s.d.'s are taken into account individually in the estimation of e.s.d.'s in distances, angles and torsion angles; correlations between e.s.d.'s in cell parameters are only used when they are defined by crystal symmetry. An approximate (isotropic) treatment of cell e.s.d.'s is used for estimating e.s.d.'s involving l.s. planes.
Refinement. Refinement of *F*^2^ against ALL reflections. The weighted *R*-factor *wR* and goodness of fit *S* are based on *F*^2^, conventional *R*-factors *R* are based on *F*, with *F* set to zero for negative *F*^2^. The threshold expression of *F*^2^ > σ(*F*^2^) is used only for calculating *R*-factors(gt) *etc*. and is not relevant to the choice of reflections for refinement. *R*-factors based on *F*^2^ are statistically about twice as large as those based on *F*, and *R*- factors based on ALL data will be even larger.

### Fractional atomic coordinates and isotropic or equivalent isotropic displacement parameters (Å^2^)

**Table:**

	*x*	*y*	*z*	*U*_iso_*/*U*_eq_	Occ. (<1)
O1	0.7476 (4)	0.54725 (14)	0.18038 (12)	0.0568 (6)	
O2	0.7536 (4)	0.14526 (14)	0.29783 (13)	0.0635 (6)	
O3	1.2666 (5)	0.48874 (17)	0.45678 (15)	0.0789 (7)	
H3	1.3738	0.4982	0.4970	0.095*	0.50
H3'	1.1026	0.4958	0.4828	0.095*	0.50
C1	0.8253 (5)	0.4491 (2)	0.24052 (17)	0.0426 (6)	
C2	0.7487 (5)	0.34076 (19)	0.23990 (17)	0.0453 (7)	
H2A	0.6341	0.3299	0.1974	0.054*	
C3	0.8453 (6)	0.2488 (2)	0.30348 (18)	0.0464 (7)	
C4	1.0120 (6)	0.2635 (2)	0.36729 (18)	0.0480 (7)	
H4A	1.0747	0.2007	0.4098	0.058*	
C5	1.0871 (5)	0.3729 (2)	0.36817 (17)	0.0425 (6)	
C6	0.9927 (5)	0.4648 (2)	0.30581 (17)	0.0438 (7)	
H6A	1.0402	0.5379	0.3069	0.053*	
C7	0.5903 (6)	0.5371 (2)	0.10822 (18)	0.0533 (7)	
H7A	0.4095	0.5024	0.1351	0.064*	
H7B	0.6854	0.4897	0.0729	0.064*	
C8	0.5585 (6)	0.6536 (2)	0.04585 (18)	0.0496 (7)	
C9	0.7312 (7)	0.6937 (3)	−0.0356 (2)	0.0754 (10)	
H9A	0.8676	0.6473	−0.0528	0.090*	
C10	0.7059 (10)	0.8012 (3)	−0.0920 (3)	0.0962 (13)	
H10A	0.8263	0.8274	−0.1467	0.115*	
C11	0.5067 (10)	0.8696 (3)	−0.0687 (3)	0.0908 (12)	
H11A	0.4883	0.9422	−0.1076	0.109*	
C12	0.3331 (9)	0.8312 (3)	0.0124 (3)	0.1023 (13)	
H12A	0.1982	0.8781	0.0297	0.123*	
C13	0.3587 (8)	0.7224 (3)	0.0687 (2)	0.0794 (10)	
H13A	0.2372	0.6959	0.1232	0.095*	
C14	0.8719 (7)	0.0468 (2)	0.3518 (2)	0.0680 (9)	
H14A	0.8331	0.0422	0.4176	0.082*	
H14B	1.0716	0.0501	0.3354	0.082*	
C15	0.7497 (7)	−0.0552 (2)	0.3328 (2)	0.0557 (8)	
C16	0.8521 (8)	−0.0914 (3)	0.2569 (2)	0.0772 (10)	
H16A	0.9985	−0.0510	0.2158	0.093*	
C17	0.7416 (8)	−0.1859 (3)	0.2410 (3)	0.0869 (11)	
H17A	0.8147	−0.2090	0.1893	0.104*	
C18	0.5285 (9)	−0.2461 (3)	0.2992 (3)	0.0790 (11)	
H18A	0.4561	−0.3106	0.2880	0.095*	
C19	0.4214 (8)	−0.2119 (3)	0.3736 (3)	0.0840 (11)	
H19A	0.2732	−0.2526	0.4136	0.101*	
C20	0.5308 (7)	−0.1165 (3)	0.3910 (2)	0.0724 (9)	
H20A	0.4552	−0.0937	0.4427	0.087*	
C21	1.2750 (6)	0.3847 (2)	0.43799 (19)	0.0533 (7)	
H21C	1.2243	0.3258	0.4958	0.064*	
H21A	1.4641	0.3721	0.4148	0.064*	

### Atomic displacement parameters (Å^2^)

**Table:**

	*U*^11^	*U*^22^	*U*^33^	*U*^12^	*U*^13^	*U*^23^
O1	0.0814 (15)	0.0381 (10)	0.0525 (11)	−0.0110 (9)	−0.0322 (10)	−0.0052 (9)
O2	0.0863 (16)	0.0319 (10)	0.0791 (14)	0.0001 (9)	−0.0387 (12)	−0.0149 (9)
O3	0.0913 (17)	0.0735 (14)	0.0969 (17)	0.0106 (12)	−0.0500 (14)	−0.0495 (13)
C1	0.0504 (17)	0.0370 (14)	0.0399 (14)	−0.0060 (12)	−0.0073 (12)	−0.0090 (12)
C2	0.0548 (18)	0.0405 (15)	0.0447 (15)	−0.0049 (12)	−0.0162 (13)	−0.0141 (12)
C3	0.0544 (18)	0.0364 (14)	0.0529 (16)	−0.0019 (12)	−0.0106 (14)	−0.0179 (13)
C4	0.0553 (18)	0.0407 (15)	0.0521 (16)	0.0023 (12)	−0.0168 (14)	−0.0156 (13)
C5	0.0382 (15)	0.0501 (16)	0.0421 (14)	−0.0022 (12)	−0.0080 (12)	−0.0166 (13)
C6	0.0480 (17)	0.0412 (15)	0.0451 (14)	−0.0094 (12)	−0.0073 (13)	−0.0162 (12)
C7	0.067 (2)	0.0495 (17)	0.0465 (15)	−0.0089 (14)	−0.0208 (14)	−0.0128 (13)
C8	0.0575 (19)	0.0477 (16)	0.0469 (16)	−0.0056 (14)	−0.0173 (14)	−0.0139 (13)
C9	0.090 (3)	0.064 (2)	0.064 (2)	−0.0044 (18)	0.0077 (19)	−0.0125 (18)
C10	0.131 (4)	0.074 (3)	0.065 (2)	−0.018 (2)	0.002 (2)	0.005 (2)
C11	0.114 (3)	0.060 (2)	0.087 (3)	−0.006 (2)	−0.039 (3)	0.010 (2)
C12	0.109 (3)	0.073 (3)	0.112 (3)	0.023 (2)	−0.015 (3)	−0.002 (2)
C13	0.082 (3)	0.073 (2)	0.070 (2)	0.014 (2)	−0.0027 (18)	0.0004 (18)
C14	0.097 (3)	0.0410 (16)	0.073 (2)	0.0056 (16)	−0.0391 (18)	−0.0153 (15)
C15	0.072 (2)	0.0330 (15)	0.0616 (19)	0.0019 (14)	−0.0210 (16)	−0.0064 (14)
C16	0.088 (3)	0.059 (2)	0.086 (2)	−0.0220 (18)	0.009 (2)	−0.0278 (18)
C17	0.093 (3)	0.072 (2)	0.109 (3)	−0.006 (2)	−0.003 (2)	−0.050 (2)
C18	0.092 (3)	0.0474 (19)	0.102 (3)	−0.0107 (19)	−0.037 (2)	−0.019 (2)
C19	0.084 (3)	0.069 (2)	0.088 (3)	−0.028 (2)	−0.020 (2)	0.001 (2)
C20	0.081 (3)	0.070 (2)	0.065 (2)	0.0024 (19)	−0.0113 (19)	−0.0148 (18)
C21	0.0524 (18)	0.0574 (17)	0.0596 (18)	0.0020 (14)	−0.0189 (14)	−0.0267 (15)

### Geometric parameters (Å, °)

**Table:**

O1—C1	1.371 (3)	C10—C11	1.354 (5)
O1—C7	1.429 (3)	C10—H10A	0.9300
O2—C3	1.374 (3)	C11—C12	1.365 (5)
O2—C14	1.416 (3)	C11—H11A	0.9300
O3—C21	1.378 (3)	C12—C13	1.381 (5)
O3—H3	0.8727	C12—H12A	0.9300
O3—H3'	0.8599	C13—H13A	0.9300
C1—C2	1.382 (3)	C14—C15	1.496 (4)
C1—C6	1.396 (3)	C14—H14A	0.9700
C2—C3	1.381 (3)	C14—H14B	0.9700
C2—H2A	0.9300	C15—C16	1.372 (4)
C3—C4	1.372 (3)	C15—C20	1.374 (4)
C4—C5	1.394 (3)	C16—C17	1.369 (4)
C4—H4A	0.9300	C16—H16A	0.9300
C5—C6	1.367 (3)	C17—C18	1.349 (5)
C5—C21	1.503 (3)	C17—H17A	0.9300
C6—H6A	0.9300	C18—C19	1.347 (5)
C7—C8	1.494 (4)	C18—H18A	0.9300
C7—H7A	0.9700	C19—C20	1.387 (4)
C7—H7B	0.9700	C19—H19A	0.9300
C8—C13	1.358 (4)	C20—H20A	0.9300
C8—C9	1.369 (4)	C21—H21C	0.9700
C9—C10	1.370 (5)	C21—H21A	0.9700
C9—H9A	0.9300		
			
C1—O1—C7	117.82 (18)	C10—C11—H11A	120.3
C3—O2—C14	117.6 (2)	C12—C11—H11A	120.3
C21—O3—H3	116.4	C11—C12—C13	119.7 (4)
C21—O3—H3'	107.9	C11—C12—H12A	120.1
H3—O3—H3'	103.4	C13—C12—H12A	120.1
O1—C1—C2	124.7 (2)	C8—C13—C12	121.0 (3)
O1—C1—C6	115.0 (2)	C8—C13—H13A	119.5
C2—C1—C6	120.3 (2)	C12—C13—H13A	119.5
C3—C2—C1	118.9 (2)	O2—C14—C15	108.6 (2)
C3—C2—H2A	120.6	O2—C14—H14A	110.0
C1—C2—H2A	120.6	C15—C14—H14A	110.0
C4—C3—O2	124.6 (2)	O2—C14—H14B	110.0
C4—C3—C2	121.2 (2)	C15—C14—H14B	110.0
O2—C3—C2	114.1 (2)	H14A—C14—H14B	108.4
C3—C4—C5	119.7 (2)	C16—C15—C20	117.6 (3)
C3—C4—H4A	120.1	C16—C15—C14	121.4 (3)
C5—C4—H4A	120.1	C20—C15—C14	121.0 (3)
C6—C5—C4	119.8 (2)	C17—C16—C15	120.9 (3)
C6—C5—C21	122.4 (2)	C17—C16—H16A	119.5
C4—C5—C21	117.8 (2)	C15—C16—H16A	119.5
C5—C6—C1	120.1 (2)	C18—C17—C16	121.0 (4)
C5—C6—H6A	119.9	C18—C17—H17A	119.5
C1—C6—H6A	119.9	C16—C17—H17A	119.5
O1—C7—C8	108.02 (19)	C19—C18—C17	119.4 (3)
O1—C7—H7A	110.1	C19—C18—H18A	120.3
C8—C7—H7A	110.1	C17—C18—H18A	120.3
O1—C7—H7B	110.1	C18—C19—C20	120.4 (3)
C8—C7—H7B	110.1	C18—C19—H19A	119.8
H7A—C7—H7B	108.4	C20—C19—H19A	119.8
C13—C8—C9	118.4 (3)	C15—C20—C19	120.6 (3)
C13—C8—C7	120.9 (3)	C15—C20—H20A	119.7
C9—C8—C7	120.8 (3)	C19—C20—H20A	119.7
C8—C9—C10	120.9 (3)	O3—C21—C5	114.2 (2)
C8—C9—H9A	119.6	O3—C21—H21C	108.7
C10—C9—H9A	119.6	C5—C21—H21C	108.7
C11—C10—C9	120.5 (4)	O3—C21—H21A	108.7
C11—C10—H10A	119.8	C5—C21—H21A	108.7
C9—C10—H10A	119.8	H21C—C21—H21A	107.6
C10—C11—C12	119.5 (4)		
			
C7—O1—C1—C2	−4.0 (4)	C7—C8—C9—C10	−178.8 (3)
C7—O1—C1—C6	176.2 (2)	C8—C9—C10—C11	−0.7 (6)
O1—C1—C2—C3	178.9 (2)	C9—C10—C11—C12	0.9 (6)
C6—C1—C2—C3	−1.3 (4)	C10—C11—C12—C13	−1.3 (6)
C14—O2—C3—C4	−10.0 (4)	C9—C8—C13—C12	−1.3 (5)
C14—O2—C3—C2	171.7 (2)	C7—C8—C13—C12	178.3 (3)
C1—C2—C3—C4	0.7 (4)	C11—C12—C13—C8	1.6 (6)
C1—C2—C3—O2	179.1 (2)	C3—O2—C14—C15	−178.2 (2)
O2—C3—C4—C5	−178.4 (2)	O2—C14—C15—C16	83.4 (4)
C2—C3—C4—C5	−0.3 (4)	O2—C14—C15—C20	−96.8 (3)
C3—C4—C5—C6	0.4 (4)	C20—C15—C16—C17	−0.9 (5)
C3—C4—C5—C21	−178.7 (2)	C14—C15—C16—C17	178.9 (3)
C4—C5—C6—C1	−1.0 (4)	C15—C16—C17—C18	0.2 (6)
C21—C5—C6—C1	178.1 (2)	C16—C17—C18—C19	0.6 (6)
O1—C1—C6—C5	−178.8 (2)	C17—C18—C19—C20	−0.7 (5)
C2—C1—C6—C5	1.5 (4)	C16—C15—C20—C19	0.8 (5)
C1—O1—C7—C8	−173.7 (2)	C14—C15—C20—C19	−179.0 (3)
O1—C7—C8—C13	−82.1 (3)	C18—C19—C20—C15	0.0 (5)
O1—C7—C8—C9	97.5 (3)	C6—C5—C21—O3	21.5 (4)
C13—C8—C9—C10	0.9 (5)	C4—C5—C21—O3	−159.4 (2)

### Hydrogen-bond geometry (Å, °)

**Table:**

*D*—H···*A*	*D*—H	H···*A*	*D*···*A*	*D*—H···*A*
O3—H3···O3^i^	0.87	1.98	2.791 (4)	155
O3—H3'···O3^ii^	0.86	1.95	2.805 (5)	179

Symmetry codes: (i) −*x*+3, −*y*+1, −*z*+1; (ii) −*x*+2, −*y*+1, −*z*+1.
